# A game study on the evolution of carbon emission reduction behavior of Chinese power enterprises

**DOI:** 10.1038/s41598-025-08645-4

**Published:** 2025-07-02

**Authors:** Yunhan Liu, Changchun Gao, Jusheng Liu

**Affiliations:** 1 Glorious Sun School of Business and Management, Donghua University, Shanghai, 200051 China; 2 School of Economics and Management, Shanghai University of Political Science and Law, Shanghai, 201701 China

**Keywords:** Carbon emission reduction, Carbon verification, Power enterprise, Collusion, Evolutionary game theory, Environmental impact, Evolutionary theory, Computational science

## Abstract

Carbon verification agencies and power enterprises play a crucial role in the process of reducing carbon emissions. Under government regulation, this paper explores the low-carbon behavior of carbon verification agencies and power enterprises, considering factors such as rewards and penalties, reputation, collusion, and costs. We first constructed a carbon emission reduction game model using evolutionary game theory and replicated dynamic equations to analyze the interactions between carbon verification agencies and power enterprises under government oversight. Subsequently, this study used theoretical derivation and numerical simulation to investigate the model’s evolution and the influence of various factors on the system’s evolution results. It is found that, firstly, the carbon emission reduction game between the carbon verification agency and the power enterprises will eventually be stabilized in two states (authentic verification and carbon emission reduction) and (fraudulent verification and no carbon emission reduction), and the specific stabilization of which state is closely related to the selection of the initial values of the parameters. Secondly, within a certain range, increasing the government’s rewards and penalties, increasing the reputation loss of carbon verification agencies and power enterprises, reducing the benefits of collusion between two parties, reducing the cost of low carbon disclosure and emission reduction of power enterprises will help the construction of a cooperative pattern of low carbon emission reduction and authentic supervision of carbon verification agencies.

## Introduction

Nowadays, the world is experiencing a low-carbon revolution driven by the increasing demand for electricity. The continued increase in fossil energy consumption has resulted in significant carbon emissions into the atmosphere^1^. The release of large quantities of carbon dioxide has led to natural phenomena such as glacier melting, biodiversity loss, and an increase in the frequency of extreme weather events^2^. To effectively mitigate the adverse effects of excessive carbon emissions, countries worldwide have initiated relevant actions. In the early years, several nations signed various carbon emission reduction agreements, including the Paris Agreement^3^ and the Kyoto Protocol^4^. Notably, the Paris Agreement mandates that all countries, not just developed ones, must take action to reduce greenhouse gas emissions. Additionally, it aims to limit the increase in global average temperature to within 2 °C above pre-industrial levels, with an aspiration to restrict it to within 1.5 °C^5^. The primary objective of the Kyoto Protocol is to achieve a 5.2% reduction in greenhouse gas emissions among developed countries between 2008 and 2012^6^.

To effectively address carbon emissions, China has proposed a series of policy measures, including strategies for carbon peaking and carbon neutrality. For instance, the Chinese government aims for carbon dioxide emissions to peak by 2030 and to achieve carbon neutrality by 2060^7^. Currently, the majority of carbon emissions in China stem from the use of fossil fuels. According to the Second Biennial Update Report on Climate Change from the People’s Republic of China (https://www.mee.gov.cn/ywdt/hjywnews/202312/W020231229717236049262.pdf), energy-related activities are the primary source of greenhouse gas emissions, accounting for approximately 86.8% of all carbon dioxide emissions in the country. Within these energy activities, carbon emissions from the power sector are a high proportion of China’s total carbon emissions. Controlling carbon emissions from the power industry is an important measure to ensure that China reaches its peak emissions as early as possible.

Currently, the power industry is a significant contributor to carbon emissions and plays a crucial role in achieving the goals of carbon peaking and carbon neutrality. The challenge of reducing carbon emissions has long been a critical concern for both academia and industry. At the technical level, carbon emission reduction encompasses various strategies, including afforestation, ocean carbon absorption, engineering storage, and other natural and artificial methods to capture and offset carbon dioxide, ultimately striving to mitigate the carbon dioxide emissions resulting from human activities. On the other hand, effectively managing enterprises and third-party carbon verification organizations to actively pursue carbon emission reductions is a critical issue, and power enterprises are no exception. Poor oversight of these organizations can result in inefficient or even ineffective carbon emission reductions. Throughout the carbon emission reduction process, it is essential for third-party verification organizations. If certain power enterprises collude with verification organizations to engage in fraudulent emission reductions, the overall efforts toward carbon emission reduction will be undermined. Therefore, it is crucial to investigate the behaviors of power enterprises and third-party carbon verification agencies to enhance the effectiveness of carbon emission reduction initiatives.

Scholars have conducted numerous studies on the reduction of carbon emissions^8^. Their research focuses on various aspects, including the low-carbon supply chain, the relationship between emerging technologies and carbon emission reduction—such as smart cities, digital technology, blockchain, e-commerce, and green innovation—carbon emission reduction efficiency prediction and optimization^9^, and the evolutionary game theory related to low-carbon initiatives. Among these areas, some researchers have examined how supply chain coordination can facilitate carbon emission reduction. For instance, Li et al.^10^ employed the Stackelberg master-slave game utility model to investigate the impact of fairness preferences on optimizing of low-carbon supply chain decision-making, including profits, levels of carbon emission reduction, warranty periods, and revenue-sharing arrangements. Du et al.^11^ utilized system dynamics to simulate the carbon emission reduction effects within the prefabricated building supply chain and discovered that subsidies play a crucial role in the carbon reduction process. Additionally, Li et al.^12^ analyzed the effects of low-carbon consumption subsidy policies and the “carbon inclusion” system on the interactions among government, enterprises, and consumers.

In addition, scholars have explored the use of emerging technologies to facilitate carbon emission reductions^13^. For instance, Hu et al.^14^ developed a system that integrates a power grid, an energy system, and a shared energy storage station to mitigate carbon emissions. Shen and Zhang^15^ posited that digital technology can indirectly lead to pollution and carbon reduction through intermediary channels, such as promoting green technology innovation, decreasing energy consumption intensity, and enhancing the energy structure. Zhu et al.^16^ investigated the impact of blockchain technology on carbon emission reductions, revealing that the implementation of blockchain does not necessarily promote reductions in carbon emissions. Zhang et al.^17^ examined carbon reduction strategies in the steel industry and concluded that the carbon metallurgical process could potentially achieve zero carbon emissions if replaced by a synergistic hydrogen-electric approach. Additionally, Zhang et al.^18^ found that the growth of e-commerce resulted in a 7.89% reduction in total CO2 emissions, equating to a per capita reduction of 1.1146 tons in selected Chinese pilot cities. Wang et al.^19^ analyzed the effects of green innovation on CO2 emissions in the context of renewable energy, carbon taxes, and GDP. Furthermore, several studies have identified additional factors that can effectively reduce carbon emissions, including low-carbon pilot city policies and smart pilot city initiatives^20,21^, advanced technologies and renewable energy sources^22^, green patents and research and development^23^, low-carbon power transitions^24^, low-carbon finance^25^, and the use of recycled building materials^26^.

As far as power enterprises are concerned, reducing low carbon emissions holds significant value for China’s overall efforts in emission reduction. At the industry level, scholars have further investigated low carbon emission reduction strategies within power enterprises. Yu et al.^27^ examined the effects of carbon emission reduction in power enterprises, focusing on both technological and structural impacts. Using the power industry as a case study, Wang et al.^22^ analyzed the factors influencing carbon dioxide emissions across various provinces in China and proposed differentiated strategies for carbon emission reduction. Wu et al.^28^ assessed the driving factors behind changes in carbon emissions in China’s power industry from 2000 to 2018. Ma et al.^29^ employed a hybrid gray model to forecast carbon emissions from China’s thermal power sector. Chen et al.^30^ investigated the regulations surrounding carbon emission quota allocation and their effects on investments in low-carbon technology within the electric power industry. Yang et al.^31^ discussed the impact of technological innovation in the power sector on carbon emissions and offered policy recommendations to foster technological advancement in China. Brauneis et al.^32^ explored the relationship between the lower bound of carbon pricing and low-carbon investments in electric utilities.

Scholars have investigated the challenges associated with reducing carbon emissions in power enterprises, primarily from a macro perspective. However, the efforts of these enterprises to reduce carbon emissions are often influenced by the behaviors of various stakeholders and other external factors. Evolutionary game theory effectively simulates the behavioral dynamics among stakeholders, allowing for an exploration of the interplay between the interests and actions of multiple parties. Consequently, researchers have employed evolutionary game theory to examine how the carbon emission reduction strategies of power enterprises are influenced by stakeholder behavior and other factors^33–35^. For example, Zhang et al.^36^ explored the game dynamics among the central government, local government, and enterprises in the process of reducing carbon emissions. They found that the central government’s environmental protection inspections drive carbon emission reductions, facilitating the formation of a stable cooperative relationship among the three parties. In response to the use of coal or electricity, Fang et al.^37^ explored the impact of taxation and subsidy policies on the long-term evolution of urban heat supply systems. Kang et al.^38^ found that controlling the price of carbon, rather than imposing a carbon cap, is an important strategy for encouraging firms to reduce their carbon emissions by constructing a game model between the government and enterprises. Wu et al.^39^ explored the low-carbon game between governments and firms, finding that government incentives, including subsidies and regulations, can effectively motivate firms to engage in low-carbon activities. Guo et al.^40^ found that different government subsidies serve distinct roles, and the rational application of these subsidies could promote the sustainable development of construction waste recycling systems and contribute to the reduction of carbon emissions.

Further, in comparison to subsidies, several studies have examined the roles of taxation, financing, and penalties in reducing carbon emissions. For instance, Chen and Hu^41^ found that taxes are more effective in motivating manufacturers to achieve carbon reductions. Zhao et al.^42^ utilized game theory to explore the importance of both internal and external financing for upstream and downstream suppliers in the context of cooperation for carbon emission reduction. Additionally, Sun et al.^43^ emphasized the importance of penalties in reducing carbon emissions, arguing that increasing penalties for passive government regulation is more effective than simply raising penalties for firms.

Existing research on government regulation, subsidies, taxes, penalties, and other factors from a microeconomic perspective explains the complex interactions among stakeholders involved in the process of reducing carbon emissions, providing valuable insights for future studies. However, the reduction of carbon emissions is a multifaceted process that encompasses various interests, and current studies fail to capture the entirety of this process. Identifying whether enterprises have engaged in carbon emissions and quantifying the amount they have emitted presents significant challenges. Consequently, governments often rely on third-party verification organizations to assess the carbon emissions of enterprises. However, this reliance can lead to potential collusion between third-party organizations and enterprises, resulting in deceptive practices that undermine government efforts to achieve carbon emission reductions. Therefore, it is crucial to investigate the behaviors of carbon verification agencies and enterprises under government regulation to effectively promote carbon emission reductions in China. Unlike existing studies, this paper develops a game-theoretic model of carbon emission reduction that considers the verification costs incurred by verification agencies, the production costs and low-carbon information disclosure costs faced by power enterprises, the reputation mechanisms of both parties, potential collusion, and the implications of government rewards and penalties, among other factors.

Distinguishing itself from existing studies, this paper presents the following contributions and innovations: (1) A novel model for reducing carbon emissions is proposed, emphasizing the relationship between carbon verification agencies and power enterprises. This model integrates a reputation mechanism along with reward and punishment systems to systematically address carbon emission reductions from a business management perspective. (2) The two-party evolutionary game method is utilized to analyze behaviors related to carbon emission reduction. This approach facilitates a clearer understanding of the complex and dynamic behaviors of enterprises in their efforts to reduce emissions, which traditional static research methods often fail to elucidate. (3) The paper investigates the collusion between carbon verification agencies and power enterprises. By modeling this collusive behavior, it underscores the detrimental effects such cooperation can have on the carbon emission reduction processes involving both enterprises and verification agencies. The findings provide valuable insights and experiences for effectively governing and mitigating collusion in this context.

The content and structure of this paper are organized as follows: Sect. 1 presents the introduction; Sect. 2 outlines the model formulation and hypotheses. Section 3 provides the model analysis, while Sect. 4 details the numerical simulation. Finally, Sect. 5 includes the discussion and conclusion.

## Model formulation and hypotheses

### Model formulation

**Fig. 1:**
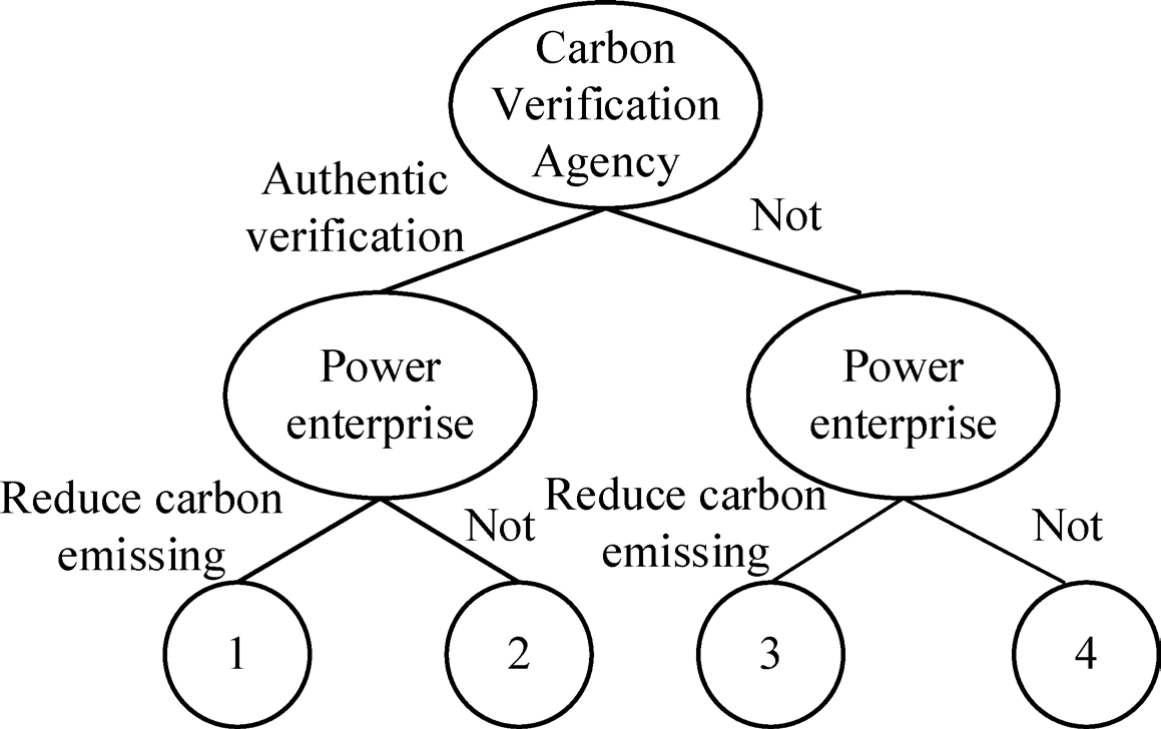
Game model diagram.

In the process of reducing carbon emissions, accurately calculating the carbon output of an enterprise poses significant challenges. It is essential for a specialized department to conduct thorough verification and subsequently report the findings to the government. This department, known as the carbon verification agency, serves as an independent third party responsible for verifying the authenticity and accuracy of carbon emission data provided by enterprises and institutions, ensuring compliance with relevant verification guidelines. To grant the carbon verification agency a degree of authority, the government mandates that it refrain from participating in or investing in enterprises, whether through equity or other means. However, during the verification process, carbon verification agencies may face dilemmas influenced by limited rationality. They might opt for an authentic verification strategy, or, swayed by potential interests, they may resort to fraudulent verification practices or even collude with enterprises to mislead the government. As the primary contributors to carbon emissions, enterprises may genuinely engage in carbon emissions or, driven by self-interest, may choose to misrepresent their emissions data to fraudulently obtain state subsidies. To effectively investigate the carbon emission reduction process and the strategic interactions between verification agencies and enterprises, this paper employs evolutionary game theory. The specific research model is illustrated in Fig. 1.

### Hypotheses

Under the framework of finite rationality, this paper develops a game model that examines the interactions between verification agencies and power enterprises within the context of government regulation. The model considers various factors, including government oversight, the reputation of verification agencies, the costs associated with verification, the production expenses, and the costs related to carbon emission reductions for power enterprises, as well as potential collusive behavior. To clarify the research problem, this paper establishes the following assumptions:

#### Hypothesis 1

Power enterprises engaged in the process of reducing carbon emissions must disclose the amount of emissions they have mitigated. In this context, third-party verification organizations often possess a significant degree of professionalism and authority. Consequently, the verification of an enterprise’s carbon emission reductions is typically conducted by third-party organizations. At this stage, these organizations may provide authentic verification or may engage in fraudulent practices. Let $\:x$ represent the proportion of authentic verification strategies chosen, while $\:1-x$ represents the proportion of fraudulent verification. Power enterprises have the option to either pursue carbon emission reductions or refrain from doing so. If they choose to engage in carbon emission reduction, they must establish a comprehensive greenhouse gas monitoring and disclosure system to effectively control carbon emissions. Additionally, they need to modify their production methods and adopt clean energy sources, which will significantly increase operational costs. Furthermore, we assume that the probability of an enterprise opting to pursue carbon emission reduction is $\:y$, while the probability of choosing not to engage in such reductions is $\:1-y$.

#### Hypothesis 2

Assuming that the cost of authentic verification is $\:{C}_{r}$ and the cost of fraudulent verification is $\:{D}_{r}$, $\:{C}_{r}>{D}_{r}$. The production cost of an electric power enterprise is $\:{C}_{e}$. If the electric utility implements measures to reduce carbon emissions, it will face additional costs, including expenses related to low-carbon information disclosure and the costs associated with reducing emissions. We can denote the cost of low-carbon information disclosure and the cost of emissions reduction as $\:{C}_{d}$.

#### Hypothesis 3

Government regulation is designed to detect instances of fraudulent verification by verification agencies and instances where enterprises fail to achieve carbon emission reductions. Under government supervision, if a verification agency conducts thorough and accurate carbon verifications, the government will provide certain incentives $\:{R}_{1}$, such as granting the agency a high level of accreditation. Likewise, if an electric power enterprise successfully reduces carbon emissions, it will also receive incentives $\:{R}_{2}$, such as the issuance of green certificates and an increased quota in the carbon market.

Conversely, if a verification agency performs fraudulent verifications while under government oversight, the government will impose penalties $\:{P}_{1}$ on the agency. These penalties may include being placed on a blacklist or having its accreditation revoked. Similarly, if enterprises do not engage in carbon emission reduction efforts, the government will penalize them through a carbon tax $\:{P}_{2}$.

#### Hypothesis 4

Enterprises and verification organizations place a high value on their reputations when it comes to reducing carbon emissions. If a verification organization conducts a fraudulent verification, it will suffer a loss of reputation, denoted as $\:{L}_{1}$. Similarly, if a power enterprise refuses to engage in carbon emission reduction, it will also incur a reputation loss, represented as $\:{L}_{2}$.

#### Hypothesis 5

The gain of the verification organization choosing authentic verification is $\:{R}_{c}$, the gain of choosing fraudulent verification is $\:{R}_{f}$. $\:{R}_{c}$ and $\:{R}_{f}$ denote the verification fees charged by the verification organization to the power enterprises when the verification organization chooses authentic verification and fraudulent verification. Compared with $\:{R}_{c}$, to save the cost of carbon emission, power enterprises will give some favors to the verification organizations to help them carry out fraudulent tests. Although the government may impose penalties on such fraudulent verifications, fraudulent verifications still have high returns compared with authentic verifications. For the sake of generality, assume that $\:{R}_{c}<{R}_{f}$.

To carry out carbon emission reduction, power enterprises may carry out technological innovations, such as improving cleaner installations and upgrading the efficiency of coal-to-power conversion, to reduce carbon dioxide emissions and achieve carbon emission reduction targets. These measures will bring some benefits to the enterprises, which is $\:{R}_{e}$. In addition, low carbon emission reduction not only requires costly technological innovation but also the construction of a low carbon disclosure system to cope with the inspection by verification agencies, for this reason, many enterprises choose not to carry out low carbon emission reduction to save costs. At this time, enterprises that do not carry out carbon emission reduction may save more costs than enterprises that carry out carbon emission reduction, which also means that they obtain more benefits, and these benefits are known as $\:{R}_{n}$. Therefore, for the sake of generality, this paper assumes that $\:{R}_{e}<{R}_{n}$.

If the enterprise does not carry out low carbon emission reduction, the power enterprise conveys certain benefits $\:F$ to the verification organization, if the verification organization carries out fraudulent verification, then the verification organization and the power enterprise form collusion, the collusion benefit is $\:S$, $\:F\:<\:S$. Collusion between power enterprises and verification agencies allows these enterprises to lower their costs associated with carbon emission reduction technologies and updates. The savings that result from this collusion can be considered benefits. Additionally, some power enterprises engage in collusive practices with verification agencies to obtain more carbon allowances from the government and to reduce their carbon tax liabilities. This type of collusion also extends to receiving government subsidies for carbon emission reductions. Thus, the concept of collusive gains is quite complex. For clarity, we will refer to these collusive benefits collectively as $\:S$. Assuming that the collusion benefit coefficient of the power enterprise is $\:\alpha\:$, then the collusion benefit coefficient of the verification organization is $\:1-\alpha\:$. Since the benefit brought by the collusion is greater than the cost of low carbon emission reduction of the power enterprise, and greater than the benefit conveyed by the power enterprise to the verification organization, therefore $\:\alpha\:S\:>\:{C}_{d}$, $\:\alpha\:S>F$.

To clearly demonstrate the meaning of each parameter, this paper further presents the parameter descriptions in tabular form as shown in Table 1.

**Table 1:** Parameter description.

Parameter	Description
$\:{C}_{r}$	Cost of authentic verification by verification organizations.
$\:{D}_{r}$	Cost of fraudulent verification by verification organizations.
$\:{C}_{e}$	Production costs of power enterprise.
$\:{C}_{d}$	Low-carbon information disclosure costs and emissions costs incurred by power enterprises.
$\:{R}_{1}$	Government incentives for verification organizations to conduct authentic verification.
$\:{R}_{2}$	Government incentives for power enterprises to make low carbon emission reductions.
$\:{P}_{1}$	Government penalties for fraudulent verification by verification organizations.
$\:{P}_{2}$	Government penalizes power enterprises for not cutting carbon emissions.
$\:{L}_{1}$	Reputational damage caused by fraudulent verification by verification organizations.
$\:{L}_{2}$	Reputational damage caused by the failure of power enterprises to carry out low carbon emissions reduction.
$\:{R}_{c}$	Benefits of authentic verification by verification organizations.
$\:{R}_{f}$	Benefits of fraudulent verification by verification organizations.
$\:{R}_{e}$	Benefits of low carbon emission reduction by power enterprises.
$\:{R}_{n}$	Benefits of not having low carbon emissions reductions in power enterprises.
$\:S$	Benefits of collusion between verification organizations and power enterprises (e.g., low carbon abatement costs saved through collusion and carbon allowances taken from the government).
$\:F$	Benefits conveyed by power enterprises to verification organizations for the purpose of collusion.
$\:\alpha\:$	Proportion of collusive gains for power enterprises.

According to the assumptions and parameter descriptions provided in Table 1, the returns for the various cases illustrated in Fig. 1 are presented in Table 2.

**Table 2:** Return of different cases in the model.

Number	Verification organization	Power enterprise	Strategy selection
①	$\:{R}_{c}-{C}_{r}+{R}_{1}$	$\:{R}_{e}-{C}_{e}-{C}_{d}+{R}_{2}$	(Authentic, reduce)
②	$\:{R}_{c}-{C}_{r}+{R}_{1}$	$\:{R}_{n}-{C}_{e}-{P}_{2}-{L}_{2}$	(Authentic, not reduce)
③	$\:{R}_{f}-{D}_{r}-{L}_{1}-{P}_{1}$	$\:{R}_{e}-{C}_{e}-{C}_{d}+{R}_{2}$	(Not authentic, reduce)
④	$\:{R}_{f}-{D}_{r}-{L}_{1}-{P}_{1}+\left(1-\alpha\:\right)S+F$	$\:{R}_{n}-{C}_{e}-{L}_{2}-{P}_{2}+\alpha\:S-F$	(Not authentic, not reduce)

According to the results presented in Table 2, the utilities for each game subject employing different strategies are as follows. The utility of a verification organization selecting the authentic verification is denoted as$\:\:\:{U}_{T}^{R}$.

1 $$\:{U}_{T}^{R}=y\left({R}_{c}-{C}_{r}+{R}_{1}\right)+\left(1-y\right){(R}_{c}-{C}_{r}+{R}_{1})$$

The utility of a verification organization that selects the fraudulent verification is denoted as $\:{U}_{T}^{N}$

2 $$\:{U}_{T}^{N}=y\left({R}_{f}-{D}_{r}-{L}_{1}-{P}_{1}\right)+\left(1-y\right)\left({R}_{f}-{D}_{r}-{L}_{1}-{P}_{1}+\left(1-\alpha\:\right)S+F\right)$$

The average utility of a verification organization choosing authentic verification and fraudulent verification is denoted as $\:\stackrel{-}{{U}_{T}}.$

3 $$\:\begin{array}{c}\stackrel{-}{{U}_{T}}=x{U}_{T}^{R}+\left(1-x\right){U}_{T}^{N}\:\end{array}$$

The utility of a power enterprise that chooses to reduce carbon emissions is represented as $\:{U}_{P}^{R}$

4 $$\:{U}_{P}^{R}=x\left({R}_{e}-{C}_{e}-{C}_{d}+{R}_{2}\right)+\left(1-x\right)\left({R}_{e}-{C}_{e}-{C}_{d}+{R}_{2}\right)$$

The utility of a power enterprise that chooses not to reduce carbon emissions is represented as

$$\:{U}_{P}^{N}$$

5 $$\:{U}_{P}^{N}=x\left({R}_{n}-{C}_{e}-{P}_{2}-{L}_{2}\right)+\left(1-x\right){(R}_{n}-{C}_{e}-{L}_{2}-{P}_{2}+\alpha\:S-F)$$

The average utility of a power enterprise that chooses to reduce and not reduce carbon emissions is denoted as $\:\stackrel{-}{{U}_{P}}$

6 $$\:\begin{array}{c}\stackrel{-}{{U}_{P}}=y{U}_{P}^{R}+\left(1-y\right){U}_{P}^{N}\:\:\:\:\:\:\:\:\:\:\:\end{array}$$

The replication dynamic equation is a dynamic differential equation that describes the changes in the selection and proportion of various strategies within the evolutionary game process^44^. The general form of the replication dynamic equation is shown in Eq. (10).

7a $$\:F\left(x\right)=\frac{dx}{dt}=x\left({m}_{n}-\stackrel{-}{m}\right),\:x\in\:\left[\text{0,1}\right]$$

7b $$\:F\left(y\right)=\frac{dy}{dt}=y\left({m}_{n}-\stackrel{-}{m}\right),y\in\:\left[\text{0,1}\right]$$

In Eq. (10), $\:x$ and $\:y$ indicate the proportion of adopting strategy $\:n$, $\:{m}_{n}$ is the payoff for adopting strategy $\:n$, $\:\stackrel{-}{m}$ denotes the average expected return when the player adopts strategy $\:n,\:\:F\left(x\right)$ and $\:F\left(y\right)$ are the replication dynamic equations.

Accordingly, the replication dynamic equations are shown in Eq. (8).

8a $$\:F\left(x,y\right)=\frac{dx}{dt}=x\left({U}_{T}^{R}-\stackrel{-}{{U}_{T}}\right)=x\left(1-x\right)({R}_{c}-{C}_{r}+{R}_{1}-{R}_{f}+{D}_{r}+{L}_{1}+{P}_{1}-\left(1-\alpha\:\right)S-F+y(\left(1-\alpha\:\right)S+F\left)\right)$$

8b $$\:H\left(x,y\right)=\frac{dy}{dt}=y\left({U}_{P}^{R}-\stackrel{-}{{U}_{P}}\right)=y\left(1-y\right)({R}_{e}-{C}_{d}+{R}_{2}-{R}_{n}+{L}_{2}+{P}_{2}-\alpha\:S+F+x\left(\alpha\:S-F\right))$$

Further, let $\:F\left(x,y\right)=0$ and $\:H\left(x,y\right)=0,$ we can get the equilibriums as$\:\:\left\{\text{0,1}\right\}$ and Eq. (9) by solving the system of two-dimensional kinetic Eq. 

9a $$\:{x}^{*}=\frac{{R}_{e}-{C}_{d}+{R}_{2}-{R}_{n}+{L}_{2}+{P}_{2}+F-\alpha\:S}{F-\alpha\:s}$$

9b $$\:{y}^{*}=\frac{{-R}_{c}+{C}_{r}-{R}_{1}+{R}_{f}-{D}_{r}-{L}_{1}-{P}_{1}+F+\left(1-\alpha\:\right)S}{F+\left(1-\alpha\:\right)S}$$

Five equilibrium points can be obtained by solving the system of two-dimensional kinetic equations, which are denoted as $\:E\left(\text{0,0}\right)$, $\:S\left(\text{0,1}\right)$, $\:W\left(\text{1,0}\right)$, $\:N\left(\text{1,1}\right)$ and $\:P({x}^{*},{y}^{*})$. Further, the Jacobian matrix of the system of kinetic equations is solved as in Eq. 10.

10 $$\:J=\left[\begin{array}{cc}\frac{\partial\:F(x,y)}{\partial\:x}&\:\frac{\partial\:F(x,y)}{\partial\:y}\\\:\frac{\partial\:H(x,y)}{\partial\:x}&\:\frac{\partial\:H(x,y)}{\partial\:y}\end{array}\right]$$

11 $$\:\frac{\partial\:F(x,y)}{\partial\:x}=(1-2x)({R}_{c}-{C}_{r}+{R}_{1}-{R}_{f}+{D}_{r}+{L}_{1}+{P}_{1}-\left(1-\alpha\:\right)S-F+y\left(\left(1-\alpha\:\right)S+F\right)$$

12 $$\:\frac{\partial\:F(x,y)}{\partial\:y}=x(1-x)(\left(1-\alpha\:\right)S+F)$$

13 $$\:\frac{\partial\:H(x,y)}{\partial\:x}=y(1-y)(\alpha\:S-F)$$

14 $$\:\frac{\partial\:H(x,y)}{\partial\:y}=(1-2y)({R}_{e}-{C}_{d}+{R}_{2}-{R}_{n}+{L}_{2}+{P}_{2}-\alpha\:S+F+x\left(\alpha\:S-F\right))$$

To calculate the determinant and trace of Jacobi matrix at each equilibrium point $\:E\left(\text{0,0}\right)$, $\:S\left(\text{0,1}\right)$, $\:W\left(\text{1,0}\right)$, $\:N\left(\text{1,1}\right)$ and $\:P({x}^{*},{y}^{*})$, the results of the Jacobi matrix at each equilibrium point are as follows:

15 $$\:{J}_{E\left(\text{0,0}\right)}=\left[\begin{array}{cc}{R}_{c}-{C}_{r}+{R}_{1}-{R}_{f}+{D}_{r}+{L}_{1}+{P}_{1}-\left(1-\alpha\:\right)S-F&\:0\\\:0&\:{R}_{e}-{C}_{d}+{R}_{2}-{R}_{n}+{L}_{2}+{P}_{2}-\alpha\:S+F\end{array}\right]$$

16 $$\:{J}_{S\left(\text{0,1}\right)}=\left[\begin{array}{cc}{R}_{c}-{C}_{r}+{R}_{1}-{R}_{f}+{D}_{r}+{L}_{1}+{P}_{1}&\:0\\\:0&\:{-(R}_{e}-{C}_{d}+{R}_{2}-{R}_{n}+{L}_{2}+{P}_{2}-\alpha\:S+F)\end{array}\right]\:$$

17 $$\:{J}_{W\left(\text{1,0}\right)}=\left[\begin{array}{cc}-({R}_{c}-{C}_{r}+{R}_{1}-{R}_{f}+{D}_{r}+{L}_{1}+{P}_{1}-\left(1-\alpha\:\right)S-F)&\:0\\\:0&\:{R}_{e}-{C}_{d}+{R}_{2}-{R}_{n}+{L}_{2}+{P}_{2}\end{array}\right]\:\:\:\:\:$$

18 $$\:{J}_{N\left(\text{1,1}\right)}=\left[\begin{array}{cc}-({R}_{c}-{C}_{r}+{R}_{1}-{R}_{f}+{D}_{r}+{L}_{1}+{P}_{1})&\:0\\\:0&\:-({R}_{e}-{C}_{d}+{R}_{2}-{R}_{n}+{L}_{2}+{P}_{2})\end{array}\right]$$

19 $$\:{J}_{P\left({x}^{*},{y}^{*}\right)}=\left[\begin{array}{cc}0&\:{x}^{*}(1-{x}^{*})(\left(1-\alpha\:\right)S+F)\\\:{y}^{*}(1-{y}^{*})(\alpha\:S-F)&\:0\end{array}\right]$$

## Model analysis

Unlike $\:\:x\:$and $\:\:y\:$, the terms$\:\:\:{x}^{*}$ and $\:{y}^{*}$ do not represent probabilities; rather, they serve as numerical notations to denote the solutions of the two-dimensional system of kinetic equations (8a) and (8b). There are no restrictions on the range of values that $\:{x}^{*}$ and $\:{y}^{*}$ can assume. Based on the values of $\:{x}^{*}$ and $\:{y}^{*}$, three cases will be examined in this study.

### Case 1

When$$\:{R}_{e}-{C}_{d}+{R}_{2}-{R}_{n}+{L}_{2}+{P}_{2}<0$$ and $$\: - R_{c} + C_{r} - R_{1} + R_{f}$$$$- D_{r} - L_{1} - P_{1} > 0$$, we find that$$\:{x}^{*}>1$$ and $$\:{y}^{*}>1$$. It can be deduced that$$\:{R}_{e}-{C}_{d}+{R}_{2}<{R}_{n}-{P}_{2}-{L}_{2}$$ and $$\:{R}_{f}-{D}_{r}-{L}_{1}-{P}_{1}>$$ $$\:{R}_{c}-{C}_{r}+{R}_{1}$$. This indicates that when the profit derived from fraudulent verification surpasses that of authentic verification, the verification agency will resort to dishonest practices, regardless of whether the power enterprise employs low-carbon or conventional emission reduction measures. Furthermore, if power enterprises realize greater profits from non-carbon emission reduction strategies compared to low-carbon approaches, they will inevitably opt for the former, irrespective of the verification agency’s choice to conduct rigorous or lenient assessments. At this point, there are four equilibrium points in the system: $$\:E\left(\text{0,0}\right)$$, $$\:S\left(\text{0,1}\right)$$, $$\:W\left(\text{1,0}\right)$$, and $$\:N\left(\text{1,1}\right)$$. Calculate the determinant and trace of each equilibrium in the Jacobian matrix, and assess the stability of each equilibrium based on its sign. The specific results are presented in Table 3.

**Table 3:** Sign of the determinant and trace of the Jacobian matrix at each equilibrium point in case 1.

Equilibrium point	Sign of determinant	Sign of trace	Stability
$\:E\left(\text{0,0}\right)$	+	−	ESS
$\:S\left(\text{0,1}\right)$	−	Uncertain	Saddle point
$\:W\left(\text{1,0}\right)$	−	Uncertain	Saddle point
$\:N\left(\text{1,1}\right)$	+	+	Unstable point

**Fig. 2:**
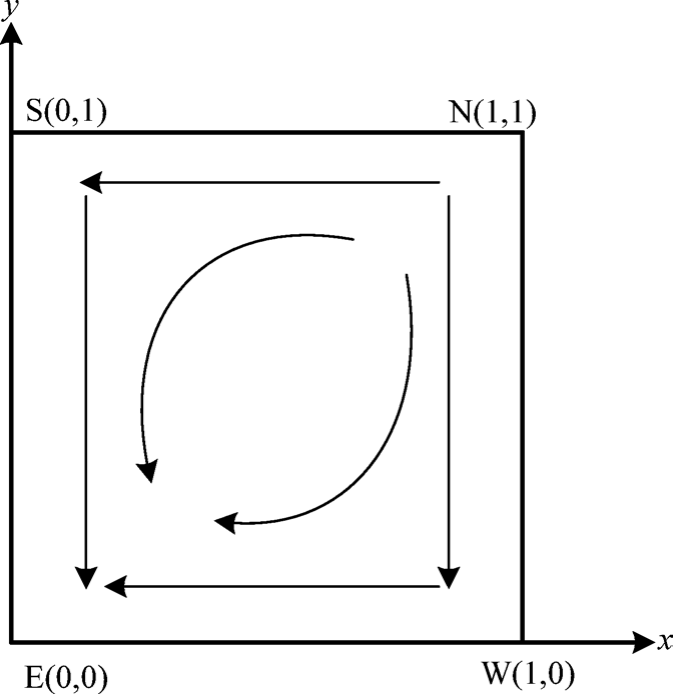
Phase diagram of case 1.

In dynamical systems, Lyapunov stability is frequently employed to assess the stability of equilibrium points. An equilibrium point is considered stable if the determinant of the Jacobian matrix, derived from a set of two-dimensional kinetic equations, is greater than 0 and the trace is less than 0. Conversely, if the determinant is greater than 0 and the trace is also greater than 0, the point is classified as unstable. If the determinant is less than 0, the point is identified as a saddle point^45^. Based on the stability analysis of equilibrium points presented in Table 3 and Lyapunov’s stability theorem, the evolutionary dynamics between the verification agency and power enterprises in Case 1 are illustrated in Fig. 2. This phase diagram effectively visualizes how the strategic choices of both entities lead to metastable states through continuous adaptation, with arrows clearly indicating the convergence trajectories toward dominant strategies under Lyapunov-stable conditions.

Phase space analysis in Case 1, as quantified in Table 3 and visualized in Fig. 2, identifies precisely one unstable point, one evolutionary stable strategy (ESS) point $\:E\left(\text{0,0}\right)$, and two saddle points $\:S\left(\text{0,1}\right)\:\:$and $\:W\left(\text{1,0}\right)$. This indicates a strong sensitivity to the initial strategy distributions between verifiers and power enterprises. Ultimately, the system’s strategy will transition from $\:N\left(\text{1,1}\right)$ to $\:E\left(\text{0,0}\right)$. Consequently, the verification organization will shift from authentic verification to fraudulent verification, while the enterprise will transition from effective carbon emission reduction to a failure in achieving low carbon emission reduction.

### Case 2

When $$\:{R}_{e}-{C}_{d}+{R}_{2}-{R}_{n}+{L}_{2}+{P}_{2}-\alpha\:S+F>0$$ and $$\: - R_{c} + C_{r} - R_{1} + R_{f} - D_{r}$$$$- L_{1} - P_{1} + F + \left( {1 - \alpha \:} \right)S < 0$$, we find that$$\:{x}^{*}<0$$ and $$\:{y}^{*}<0$$. It can be deduced that $$\:R_{e} - C_{d} + R_{2} > R_{n}$$$$- L_{2} - P_{2} + \alpha \:S - F$$ and $$\:R_{f} - D_{r} - L_{1} - P_{1} + F$$$$+ \left( {1 - \alpha \:} \right)S < R_{c} - C_{r} + R_{1}$$. This indicates that when the profits derived from authentic verification by the agency exceed those from fraudulent practices, the agency will inevitably prioritize rigorous assessments, regardless of whether power enterprises adopt low-carbon or conventional strategies. Similarly, if enterprises realize higher profits from low-carbon initiatives compared to non-low-carbon approaches, they will clearly opt for the former, irrespective of the verification agency’s decision between strict compliance checks and compromised evaluations. At this point, there are four equilibrium points in the system: $$\:E\left(\text{0,0}\right)$$, $$\:S\left(\text{0,1}\right)$$, $$\:W\left(\text{1,0}\right)$$, and $$\:N\left(\text{1,1}\right)$$. Calculate the determinant and trace of each equilibrium in the Jacobian matrix, and determine the stability of each equilibrium based on its sign. The specific results are presented in Table 4.

**Table 4:** Sign of the determinant and trace of the Jacobian matrix at each equilibrium point in case 2.

Equilibrium point	Sign of determinant	Sign of trace	Stability
$\:E\left(\text{0,0}\right)$	+	+	Unstable point
$\:S\left(\text{0,1}\right)$	−	Uncertain	Saddle point
$\:W\left(\text{1,0}\right)$	−	Uncertain	Saddle point
$\:N\left(\text{1,1}\right)$	+	−	ESS

**Fig. 3:**
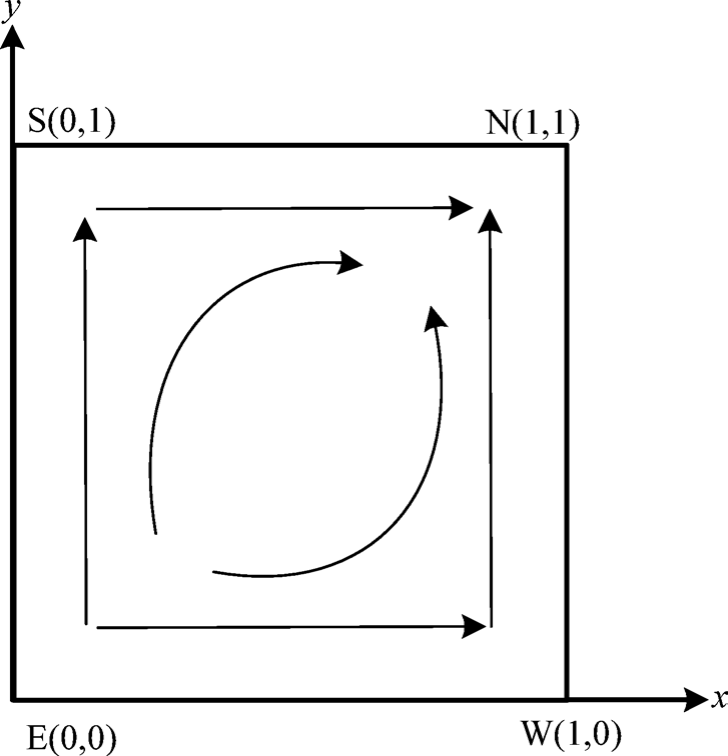
Phase diagram of case 2.

Based on the stability analysis of equilibrium points and Lyapunov’s stability theorem, the evolutionary dynamics between the verification agency and power enterprises in Case 2 are depicted in Fig. 3.

As illustrated in Table 4; Fig. 3, there are four equilibrium points in Case 2. According to Lyapunov stability theory, we identify one unstable point, $\:E\left(\text{0,0}\right)$, one point of evolutionary stabilization, $\:N\left(\text{1,1}\right)$, and two saddle points, $\:S\left(\text{0,1}\right)$ and $\:W\left(\text{1,0}\right)$. The system’s final strategy will transition from $\:E\left(\text{0,0}\right)$ to $\:N\left(\text{1,1}\right)$. This indicates that the verification organization will move from fraudulent verification to authentic verification, while the enterprise will shift from a lack of low carbon emission reduction to implementing low carbon emission reduction strategies.

### Case 3

When$$\:{0<R}_{e}-{C}_{d}+{R}_{2}-{R}_{n}+{L}_{2}+{P}_{2}<\alpha\:S-F$$ and $$- \left( {1 - \alpha \:} \right)S - F < - R_{c} + C_{r}$$$$- R_{1} + R_{f} - D_{r} - L_{1} - P_{1} < 0$$, we find that$$\:0<{x}^{*}<1$$ and $$\:0<{y}^{*}<1$$. It can be deduced that $$\:{R}_{n}-{C}_{e}-{L}_{2}-{P}_{2}$$+ $$\:\alpha\:S-F$$ >$$\:{R}_{e}-{C}_{e}-{C}_{d}+{R}_{2}>{R}_{n}-{C}_{e}-{L}_{2}-{P}_{2}$$ and $$R_{f} - D_{r} - L_{1} - P_{1} + \left( {1 - \alpha \:} \right)S - F$$$$> R_{c} - C_{r} + R_{1} > R_{f} - D_{r} - L_{1} - P_{1}$$. This indicates that when the verification agency opts for authentic verification, the power enterprise will actively pursue reductions in carbon emissions. Conversely, when the verification agency selects fraudulent verification, the power enterprise will refrain from implementing such reductions. When power enterprises choose to pursue carbon emission reductions, the verification agency will opt for authentic verification; conversely, when power enterprises decide against pursuing these reductions, the verification agency will select fraudulent verification. Therefore, the strategic choices of both the verification agency and the power enterprises are not static; rather, they will evolve in response to the strategic decisions made by both parties. At this point, there are five equilibrium points in the system: $$\:E\left(\text{0,0}\right)$$, $$\:S\left(\text{0,1}\right)$$, $$\:W\left(\text{1,0}\right)$$, $$\:N\left(\text{1,1}\right)$$, and $$\:P({x}^{*},{y}^{*})$$. Calculate the determinant and trace of each equilibrium in the Jacobian matrix and determine the stability of each equilibrium based on its sign. The specific results are shown in Table 5.

**Table 5:** Sign of the determinant and trace of the Jacobian matrix at each equilibrium point in case 3.

Equilibrium point	Sign of determinant	Sign of trace	Stability
$\:E\left(\text{0,0}\right)$	+	−	ESS
$\:S\left(\text{0,1}\right)$	+	+	Unstable point
$\:W\left(\text{1,0}\right)$	+	+	Unstable point
$\:N\left(\text{1,1}\right)$	+	−	ESS
$\:P({x}^{*},{y}^{*})$	−	0	Saddle point

**Fig. 4:**
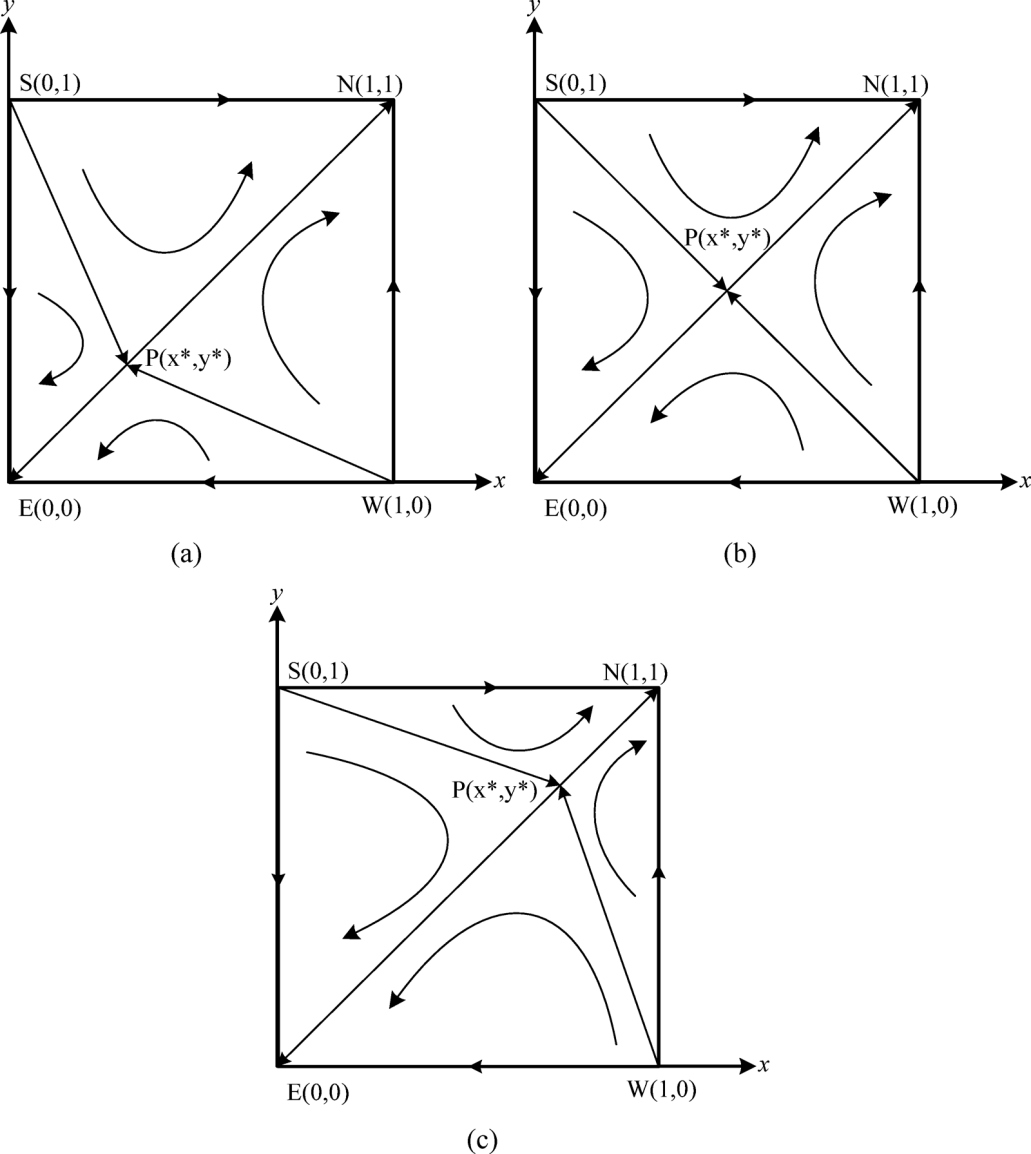
Phase diagram of case 3. $\:\left(\mathbf{a}\right)\:0<{x}^{*}<\frac{1}{2},\:0<{y}^{*}<\frac{1}{2}\:\left(\mathbf{b}\right){x}^{*}=\frac{1}{2},\:{y}^{*}=\frac{1}{2}$. $\:\left(\mathbf{c}\right)\:\frac{1}{2}<{x}^{*}<1,\:\frac{1}{2}<{y}^{*}<1$.

Based on the stability analysis of equilibrium points and Lyapunov’s stability theorem, the evolutionary dynamics between the verification agency and power enterprises in Case 3 are depicted in Fig. 4.

In Case 3, as illustrated in Table 5; Fig. 4, there are five equilibrium points within the system: two evolutionary stabilization strategy points, $\:E\left(\text{0,0}\right)$ and $\:N\left(\text{1,1}\right)$; two unstable points, $\:S\left(\text{0,1}\right)$ and $\:W\left(\text{1,0}\right)$; and one saddle point, $\:P({x}^{*},{y}^{*})$. At this stage, the strategies within the system will transition from unstable points to stable points, with the eventual stabilization point depending on the values of$\:{x}^{*}$ and $\:{y}^{*}$. As demonstrated in Fig. 4, the equilibrium strategy to which the system converges is influenced by the areas of regions$\:ESPW$ and $\:NSPW$. When the area of region $\:ESPW$ exceeds that of $\:NSPW$, the system asymptotically stabilizes at equilibrium point $\:E\left(\text{0,0}\right)$. Conversely, when the area of region $\:NSPW$ surpasses that of $\:ESPW$, the system asymptotically stabilizes at equilibrium point $\:N\left(\text{1,1}\right)$. In cases where the areas of regions $\:ESPW$ and $\:NSPW$ are equal, the system may asymptotically stabilize at either equilibrium point $\:E\left(\text{0,0}\right)\:$or $\:N\left(\text{1,1}\right)$. As shown in Fig. 4a, if $\:{0<x}^{*}<1/2$ and $\:{0<y}^{*}<1/2$, the system will stabilize at $\:N\left(\text{1,1}\right)$. In Fig. 4b, if$\:{x}^{*}=1/2$ and $\:{y}^{*}=1/2$, the system will stabilize at either$\:E\left(\text{0,0}\right)$ or $\:N\left(\text{1,1}\right)$. In Fig. 4c, if$\:{1/2<x}^{*}<1$ and $\:{1/2<y}^{*}<1$, the system will stabilize at $\:E\left(\text{0,0}\right)$.

## Numerical simulation

### Phase diagram simulation

To investigate the evolution of strategies employed by verification organizations and power enterprises within the system, this paper conducts numerical simulations of three game scenarios by selecting relevant parameters to validate the accuracy of the theoretical derivations. Data released by China’s Ministry of Ecology and Environment indicates that certain enterprises and organizations have falsified carbon data. Reports suggest that enterprises can reduce their reported carbon emissions by 10–30% through collusion with carbon verification organizations. This manipulation can result in savings exceeding 50 million yuan on carbon emission reduction costs for these enterprises (https://www.cdmfund.org/30571.html). Furthermore, according to the Ministry’s Carbon Emission Trading Management Measures (Trial), the penalties for falsifying carbon data range from 10,000 to 30,000 yuan. Therefore, penalties $\:{P}_{1}$ and $\:{P}_{2}$ should be set at a minimum of 10,000 yuan. Additionally, power enterprises that are unwilling to reduce low carbon emissions through technological and equipment upgrades have the option to purchase carbon quotas as an alternative. As of April 24, 2024, the market price for carbon was 100.59 yuan/ton (https://iigf.cufe.edu.cn/info/1019/8996.htm). If these power enterprises consume between 200 and 300 tons of coal daily, the cost of the purchased carbon quotas—which serve as a way to offset their carbon emission reductions—would range from 20,000 to 30,000 yuan. Therefore, $\:{C}_{d}$ can be set at 20,000 yuan. Furthermore, according to the carbon verification tender notice published by the Beijing Finance Bureau, the cost of a single carbon verification is around 30,000 to 50,000 yuan per verification. Thus, $\:{C}_{r}$ and $\:{D}_{r}$ can be set at 50,000 and 30,000 yuan. According to the actual situation and the China Environmental Statistics Yearbook 2023, and combined with relevant research^26,40,46^, the values of other parameters are selected as follows. The following parameters involving money are all in units of 10,000 yuan.

**Fig. 5:**
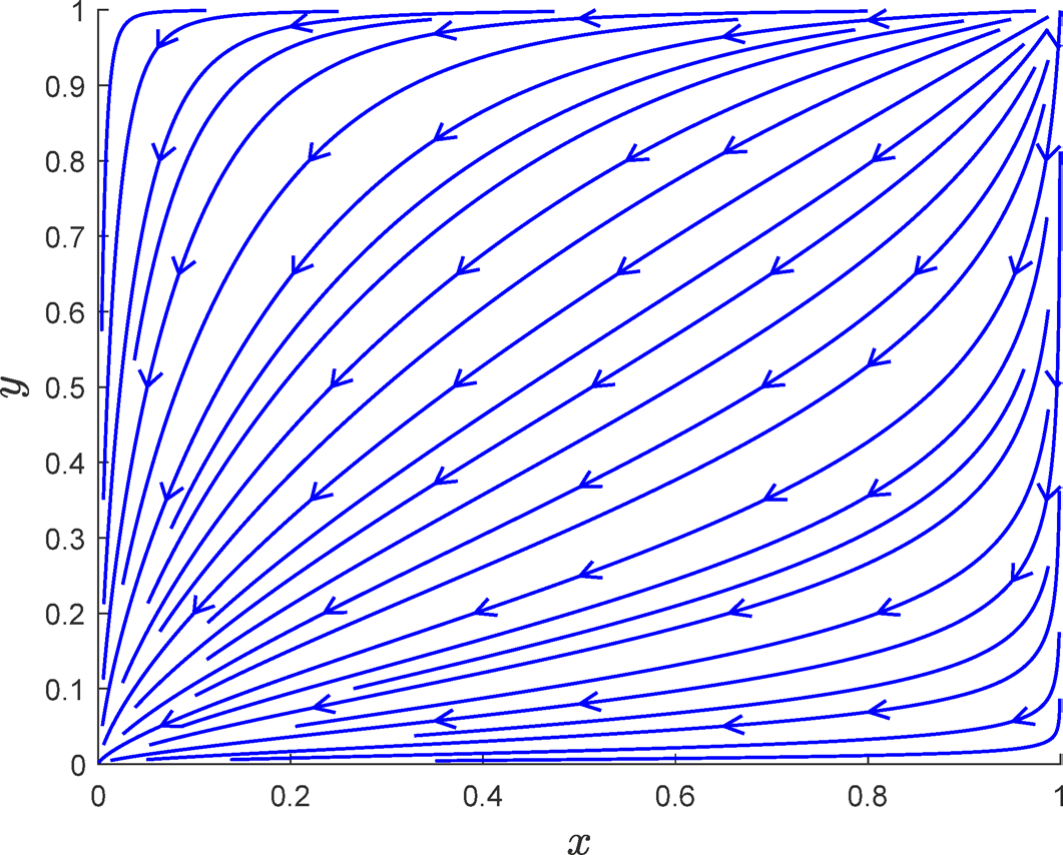
Phase diagram simulation of case 1.

In Case 1, the parameters selected for this study are as follows: $\:{C}_{r}=5$, $\:{D}_{r}=3$, $\:{R}_{e}=8$, $\:{R}_{n}=25$, $\:{R}_{1}=2$, $\:{R}_{2}=4$, $\:{R}_{c}=8$, $\:{R}_{f}=20$, $\:{L}_{1}=2$, $\:{L}_{2}=2$, $\:{P}_{1}=1$, $\:{P}_{2}=4$, $\:{C}_{d}=2$, $\:a=0.5$, $\:S=40$, and $\:F=8$. These parameters have been chosen to meet the requirements of Case 1, and the phase diagram simulation for this case is illustrated in Fig. 5.

As shown in Fig. 5, it can be seen that in Case 1, when $\:{R}_{e}-{C}_{d}+{R}_{2}-{R}_{n}+{L}_{2}+{P}_{2}<0$ and $\:{-R}_{c}+{C}_{r}-{R}_{1}+{R}_{f}-{D}_{r}-{L}_{1}-{P}_{1}>0$, the final evolution of the system will result in a shift from authentic verification and carbon reduction to a state of fraudulent verification and no carbon reduction. This outcome aligns with the earlier theoretical derivation.

**Fig. 6:**
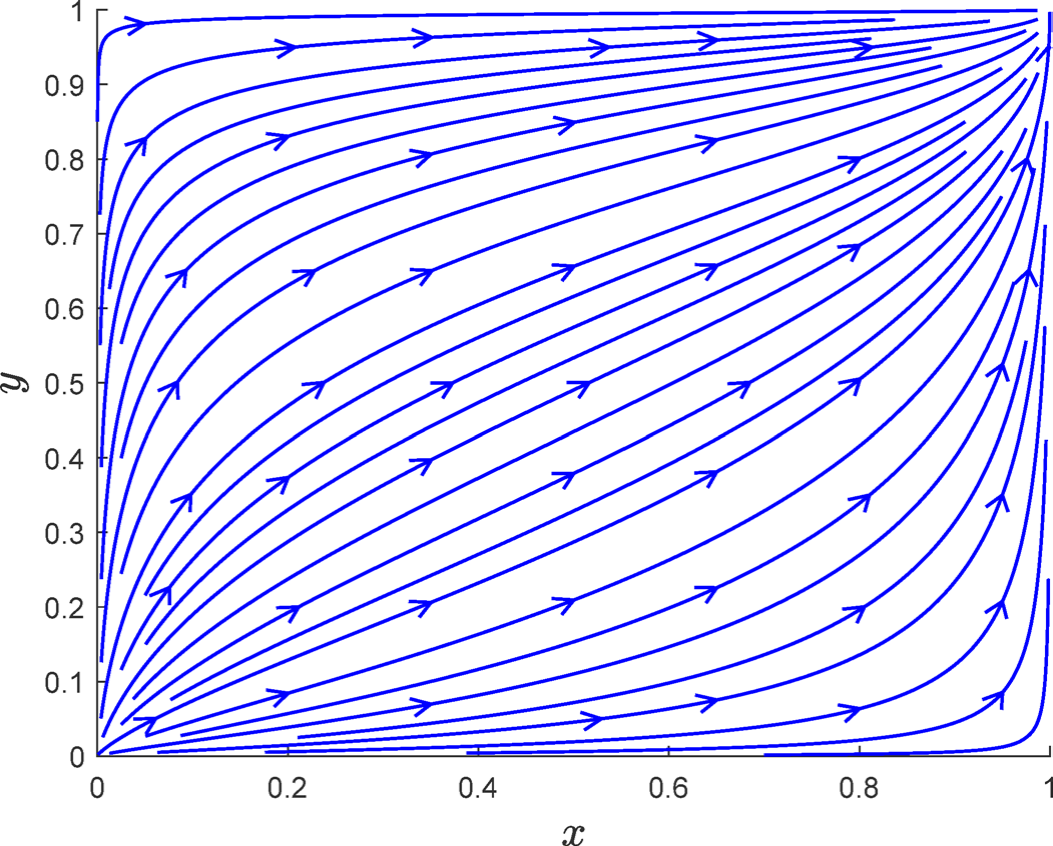
Phase diagram simulation of case 2.

In Case 2, the parameters of this paper are selected as follows: $\:{C}_{r}=5$, $\:{D}_{r}=3$, $\:{R}_{e}=8$, $\:{R}_{n}=12$, $\:{R}_{1}=2$, $\:{R}_{2}=4$, $\:{R}_{c}=8$, $\:{R}_{f}=20$, $\:{L}_{1}=2$, $\:{L}_{2}=2$, $\:{P}_{1}=30$, $\:{P}_{2}=10$, $\:{C}_{d}=2$, $\:a=0.5$, $\:S=20$, and $\:F=8$. These parameters are chosen to satisfy the conditions of Case 2 and the phase diagram simulation for this case is shown in Fig. 6.

As shown in Fig. 6, when $\:{R}_{e}-{C}_{d}+{R}_{2}-{R}_{n}+{L}_{2}+{P}_{2}-\alpha\:S+F>0$ and $\:{-R}_{c}+{C}_{r}-{R}_{1}+{R}_{f}-{D}_{r}-{L}_{1}-{P}_{1}+F+\left(1-\alpha\:\right)S<0$, the final evolution of the system will result in a shift from fraudulent verification and no carbon reduction to an authentic verification and carbon reduction state. This outcome aligns with the earlier theoretical derivation.

**Fig. 7:**
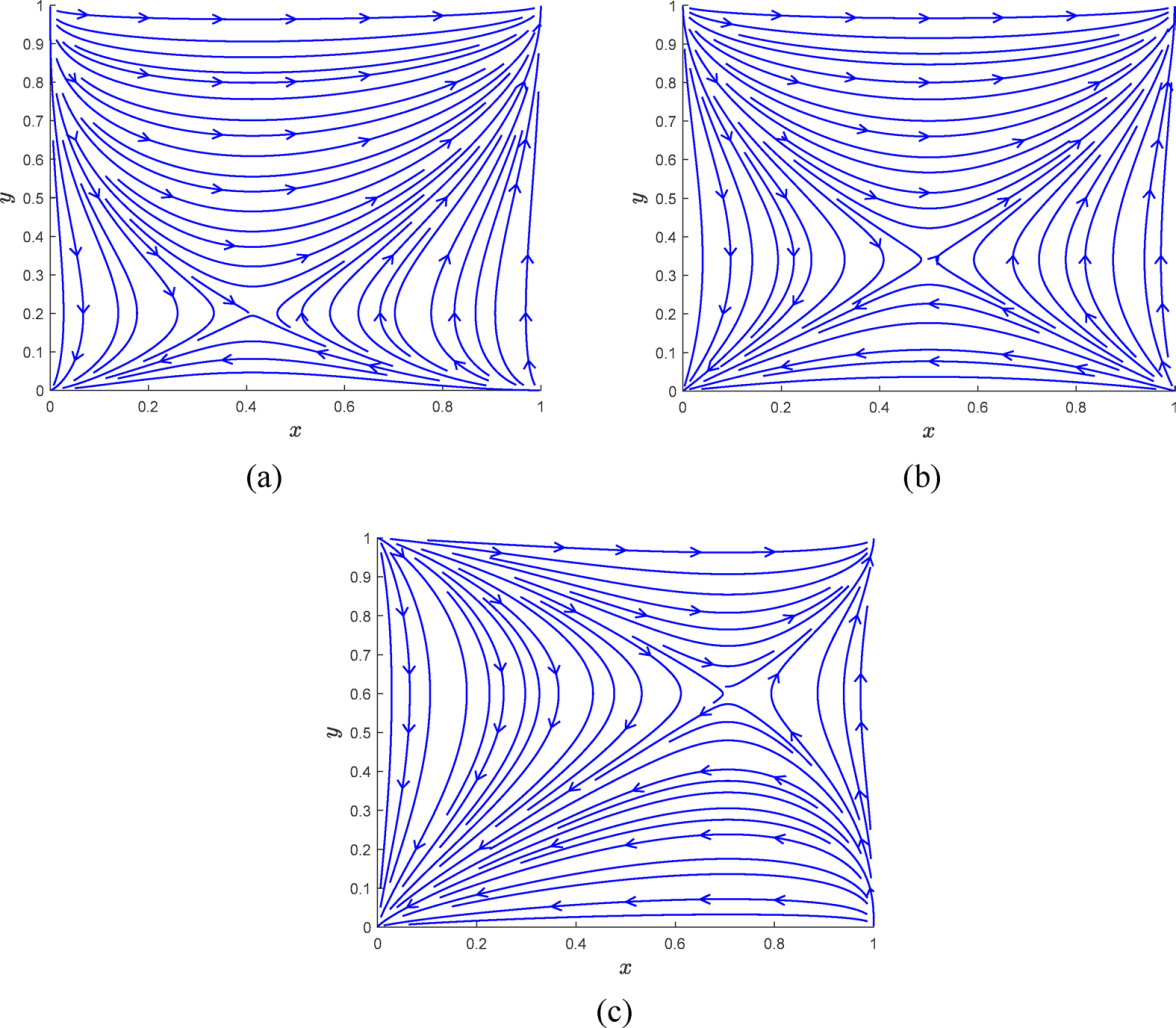
Phase diagram simulation of case 3. $\:\left(\mathbf{a}\right)\:0<{x}^{*}<\frac{1}{2},\:0<{y}^{*}<\frac{1}{2}\:\left(\mathbf{b}\right){x}^{*}=\frac{1}{2},\:{y}^{*}=\frac{1}{2}$. $\:\left(\mathbf{c}\right)\:\frac{1}{2}<{x}^{*}<1,\:\frac{1}{2}<{y}^{*}<1$

In Case 3, the parameters selected for this study are as follows: $\:{C}_{r}=5$, $\:{D}_{r}=3$, $\:{R}_{e}=8$, $\:{R}_{n}=12$, $\:{R}_{1}=2$, $\:{R}_{2}=4$, $\:{R}_{c}=8$, $\:{R}_{f}=20$, $\:{L}_{1}=2$, $\:{L}_{2}=2$, $\:{P}_{1}=30$, $\:{P}_{2}=10$, $\:{C}_{d}=2$, $\:a=0.5$, $\:S=50$, and $\:F=8$. These parameters are chosen to meet the conditions of Case 3, and the phase diagram simulation for this case is illustrated in Fig. 7. When $\:{P}_{1}=30$ and $\:{P}_{2}=10$, the values of$\:\:{x}^{*}$ and $\:{y}^{*}$ satisfy the conditions $\:0<{x}^{*}<\frac{1}{2}$ and $\:0<{y}^{*}<\frac{1}{2}$ ; the corresponding phase diagram simulation is shown in Fig. 7a. When $\:{P}_{1}=26.5$ and $\:{P}_{2}=8.5$, the values of $\:\:{x}^{*}$ and $\:{y}^{*}$ satisfy the conditions $\:{x}^{*}=\frac{1}{2}$ and $\:{y}^{*}=\frac{1}{2}$, the phase diagram simulation for this case is depicted in Fig. 7b. Finally, when $\:{P}_{1}=20$ and $\:{P}_{2}=5$, the values of$\:\:{x}^{*}$ and $\:{y}^{*}$ satisfy the conditions $\:\frac{1}{2}<{x}^{*}<1$ and $\:\frac{1}{2}$ < $\:{y}^{*}$ < 1, the phase diagram simulation for this case is depicted in Fig. 7c.

As shown in Fig. 7, when $\:0<{x}^{*}<\frac{1}{2}$ and $\:0<{y}^{*}<\frac{1}{2}$, the final evolution of the system will result in a shift to a authentic verification and carbon reduction state; when $\:{x}^{*}=\frac{1}{2}$ and $\:{y}^{*}=\frac{1}{2}$, the final evolution of the system will result in a shift to a authentic verification and carbon reduction state or a state of fraudulent verification and no carbon reduction. When $\:\frac{1}{2}<{x}^{*}<1$ and $\:\frac{1}{2}$<$\:{y}^{*}$<1, the final evolution of the system will result in a shift to a state of fraudulent verification and no carbon reduction. This result is consistent with the previous theoretical derivation.

### Influence factor simulation

Taking Case 3 as an example, this study examines the factors that influence verification organizations in detecting and power enterprises in implementing carbon emission reduction strategies. To investigate the role of these influencing factors, this paper employs numerical analysis methods for simulation. The relevant simulation scenarios are outlined as follows.

#### The influence of $\:{\varvec{R}}_{1}$ and $\:{\varvec{R}}_{2}$

**Fig. 8:**
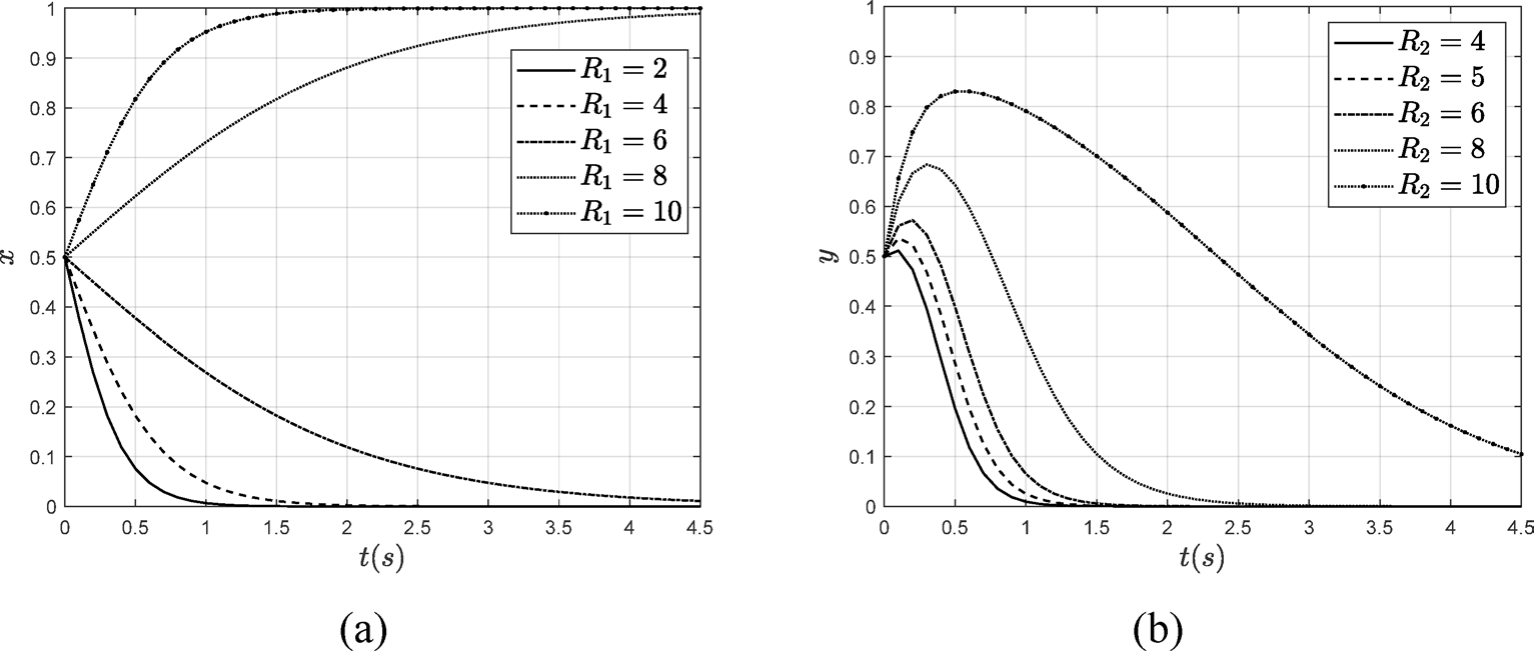
The influence of $\:{R}_{1}$ and $\:{R}_{2}$ on the system evolution results. (**a**) The influence of $\:{R}_{1}$ on the $\:x$ (**b**) The influence of $\:{R}_{2}$ on the $\:y$.

To investigate the impact of $\:{R}_{1}$ and $\:{R}_{2}$on the evolution of the system, while keeping all other parameters constant, $\:{R}_{1}$ was increased from 2 to 10 and $\:{R}_{2}$was increased from 4 to 10. The results of this analysis are presented in Fig. 8.

As illustrated in Fig. 8, when $\:{R}_{1}$ increases from 2 to 6, it is evident that the verification organizations tend to opt for fraudulent verification. As the incentives for these organizations to conduct rigorous testing increase, the rate at which they choose to engage in fraudulent verification gradually declines. Conversely, when government incentives for verification organizations rise from 8 to 10, these organizations are more likely to select authentic verification, and the rate at which they choose authentic verification accelerates as the incentives increase.

When a power enterprise implements carbon emission reduction strategies, the government provides certain incentives, such as granting carbon market quotas. As the government’s rewards for power enterprises increase from 4 to 10, the percentage of power enterprises opting not to pursue carbon emission reduction decreases significantly. With higher incentives, both the rate and proportion of power enterprises adopting carbon emission reduction strategies will continue to rise. Therefore, rewards serve as a crucial management tool in the government’s efforts to govern carbon emission reduction behaviors.

#### The influence of $\:{\varvec{L}}_{1}$ and $\:{\varvec{L}}_{2}$

**Fig. 9:**
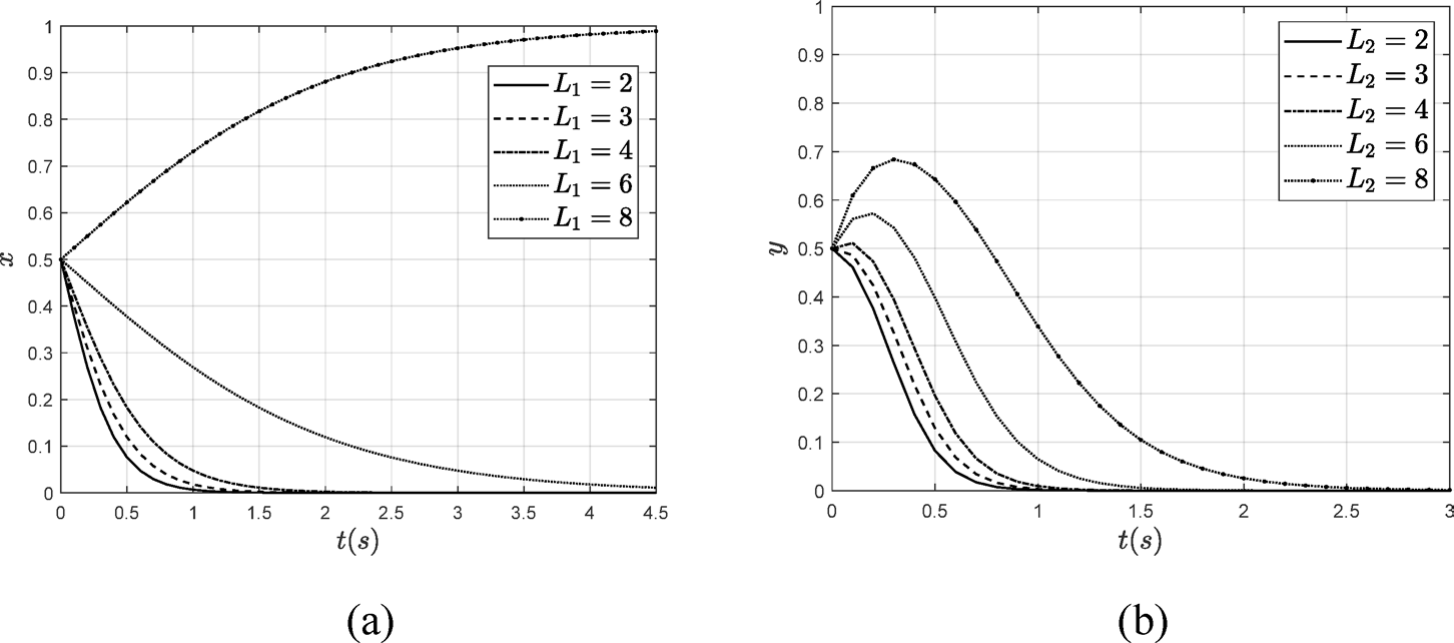
The influence of $\:{L}_{1}$ and $\:{L}_{2}$ on the system evolution results. (**a**) The influence of$\:{L}_{1}$ on the$\:x$ (**b**) The influence of $\:{L}_{2}$ on the $\:y$.

Reputation, as a crucial factor, significantly impacts an individual’s behavior. This paper examines the influence of reputation on the behavior of verification organizations and power enterprises by increasing the loss of reputation from 2 to 8 for these entities, respectively, while keeping other parameters constant. The influence of $\:{L}_{1}$ and $\:{L}_{2}$ on the system’s evolution results are illustrated in Fig. 9.

As illustrated in Fig. 9, when $\:{L}_{1}$ and $\:{L}_{2}$ increase from 2 to 8, the verification organization gradually transitions from fraudulent verification to authentic verification. Notably, when $\:{L}_{1}$ reaches 8, the verification organization opts for authentic verification. Similarly, as $\:{L}_{2}$ increases from 2 to 8, although the power enterprise initially does not adopt low-carbon strategies, the increasing reputation loss causes the rate at which the power enterprise avoids low-carbon strategies to decelerate, eventually leading to a preference for low-carbon strategies. Therefore, reputation, as an intangible asset for enterprises, plays a crucial role in shaping their behavioral choices and decisions.

#### The influence of $\:{\varvec{P}}_{1}$ and $\:{\varvec{P}}_{2}$

**Fig. 10:**
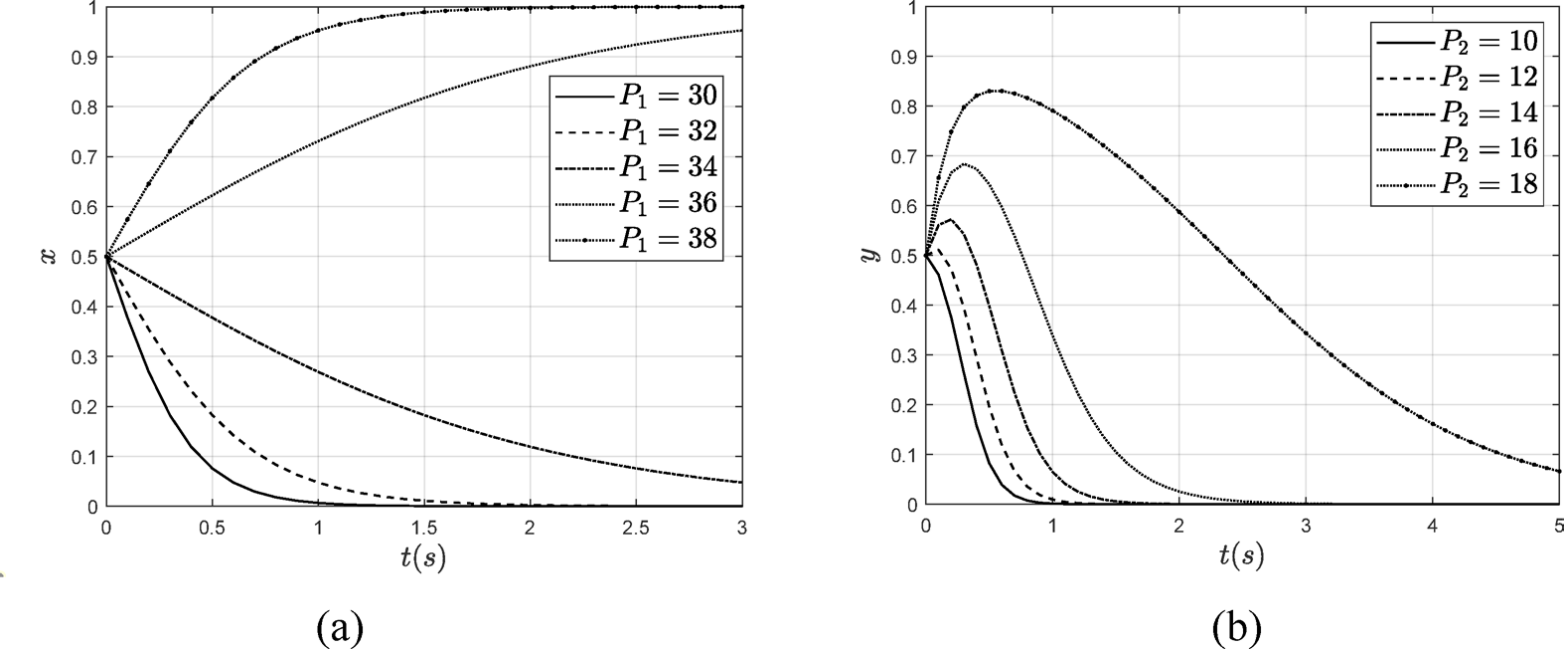
The influence of $\:{P}_{1}$ and $\:{P}_{2}$ on the system evolution results. (**a**) The influence of $\:{P}_{1}$ on the $\:x$ (**b**) The influence of $\:{P}_{2}$ on the $\:y$.

To investigate the effect of punishment on the evolution of system strategies, this paper assigns values to $\:{P}_{1}$ and $\:{P}_{2}$, where $\:{P}_{1}$ increases from 30 to 38 and $\:{P}_{2}$ increases from 10 to 18. The influence of $\:{P}_{1}$ and $\:{P}_{2}$ on the system evolution results is illustrated in Fig. 10.

As illustrated in Fig. 10, when $\:{P}_{1}$ increases from 30 to 38, the verification organization opts for and stabilizes at authentic verification. This indicates that an increase in penalties plays a significant role in regulating institutional behavior. Similarly, when $\:{P}_{2}$ rises from 10 to 18, the duration of steps that a power enterprise takes to select a non-carbon emission reduction strategy becomes progressively longer, and the rate of change slows down. If power enterprises fail to implement carbon emission reduction measures, the government can increase penalties, such as raising the carbon tax. This approach effectively encourages enterprises to reduce the likelihood of not adopting carbon emission reduction strategies, thereby leading them to favor carbon emission reduction initiatives.

#### The influence of $\:\varvec{S}$

**Fig. 11:**
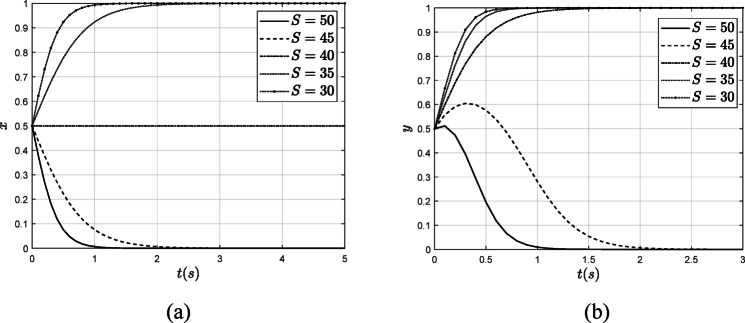
The influence of $\:S$ on the system evolution results. (**a**) The influence of $\:S$ on the $\:x$ (**b**) The influence of $\:S$ on the $\:y$.

Keeping all other parameters constant, this study explores the impact of collusion benefits on system evolution results by varying the magnitude of collusion benefits between the verification organization and the power enterprise, as illustrated in Fig. 11.

As illustrated in Fig. 11, when the benefits of collusion between verification organizations and power enterprises decrease from 50 to 40, the verification organization will transition from fraudulent verification to authentic verification. At this point, 40 serves as the threshold for collusion benefits; when these benefits fall below 40, the verification organization opts for authentic verification, whereas if they exceed 40, it will continue to engage in fraudulent verification. The lower the collusion benefits, the more rapidly the verification organization will adopt authentic verification. Similarly, as collusion benefits decline from 50 to 30, the power enterprise will shift from a no-low-carbon strategy to a low-carbon strategy, with the transition to a low-carbon approach accelerating as collusion benefits diminish. Consequently, collusion benefits are a critical factor influencing the interactions between the two parties. In the context of government governance, it is essential to focus on combating collusion, reducing collusion benefits, and systematically promoting carbon emission reductions.

#### The influence of $\:{\varvec{C}}_{\varvec{d}}$

**Fig. 12:**
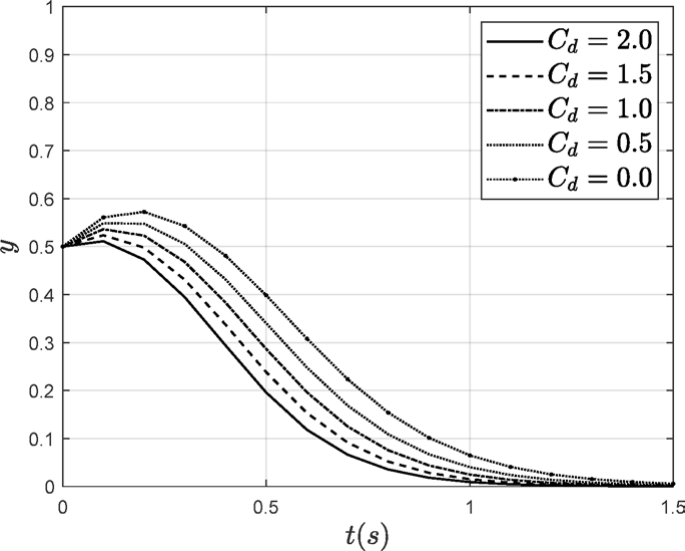
The influence of $\:{C}_{d}$ on the power enterprise evolution results.

In the process of reducing carbon emissions, the cost of emission reduction significantly influences the behavior of organizations engaged in this effort. To investigate this impact, we maintained all other parameters constant while decreasing the abatement cost from 2.0 to 0.0. This analysis aims to explore how changes in abatement costs affect the carbon emission reduction behavior of power enterprises. The specific results are illustrated in Fig. 12.

As illustrated in Fig. 12, when the cost of emission reduction $\:{C}_{d}$ decreases from 2.0 to 0.0, it is evident that although power enterprises initially opt for non-low-carbon strategies, the reduction in low-carbon abatement costs encourages these enterprises to increase their reliance on non-low-carbon strategies. Simultaneously, the rate at which they choose non-low-carbon strategies slows down. This indicates that lowering the cost of carbon emission reduction can effectively motivate power enterprises to engage in carbon emission reduction activities.

## Discussion and conclusion

### Discussion

Carbon emission reduction is a priority for governments at all levels in China. To mitigate carbon emissions, verification organizations operate as independent third-party entities that assess the emissions of enterprises. However, existing studies have largely overlooked the dynamics of the interactions between carbon verification agencies and power enterprises, as well as the challenges to carbon emission reduction posed by collusive behavior between these two parties. To investigate the behavior of carbon emission reduction between carbon verification agencies and power enterprises under government regulation, this paper constructs a carbon emission reduction game model utilizing evolutionary game theory. The model considers various factors, including government rewards and penalties, costs, reputation, collusion, and others. The carbon verification agency can choose between authentic verification and fraudulent verification, while the power enterprise can opt for either a low-carbon or non-low-carbon production strategy. The findings indicate that increasing the rewards and penalties imposed by the government on both the verification agency and the power enterprises, reducing the reputation loss for both parties, diminishing the benefits derived from collusion, and lowering the costs associated with low-carbon information disclosure and abatement for power enterprises will encourage the verification agency to adopt an authentic regulation strategy and prompt power enterprises to implement a low-carbon abatement strategy.

This study aligns with several research findings^41^, which highlight the importance of increasing government incentives and penalties for institutions and enterprises that contribute to carbon emission reductions^47^. However, the government can employ various methods to incentivize and penalize, not solely relying on financial measures. For instance, high-carbon market quotas could be allocated to enterprises and institutions that actively engage in carbon emission reduction efforts^48,49^. Regarding penalties, a carbon tax can serve as an effective punitive measure. Additionally, implementing reputation mechanisms, such as establishing a blacklist of non-compliant enterprises and organizations, can further motivate these entities to pursue carbon emission reductions. Finally, attention must be given to the potential collusion between carbon verification agencies and enterprises, and efforts to combat such collusion should be strengthened to ensure the effective management of carbon emissions.

Based on the results of this study, we offer several actionable insights for policymakers and corporate decision-makers. First, our findings underscore the necessity of implementing non-monetary incentives and disincentives, such as carbon market quotas and carbon taxes, which can serve as agency-imposed rewards and penalties to encourage and regulate low-carbon enterprises, motivating them to align with low-carbon objectives. Second, a reputation mechanism can be employed as an effective tool to regulate collusive behavior. For instance, a public blacklist of colluding entities can amplify the hidden costs associated with non-compliance. Third, anti-collusion measures—including blockchain-enabled data traceability and cross-validation protocols—can be integrated into corporate compliance frameworks to mitigate the risk of fraud. Finally, reducing the costs associated with decarbonizing businesses through standardized carbon accounting tools and shared technology platforms offers a strategic pathway for resource allocation. These steps bridge the gap between model-based insights and real-world organizational decision-making by translating theoretical dynamics into practical governance tools, enabling policymakers to design targeted regulations and allowing enterprises to balance compliance costs with long-term reputational benefits.

### Theoretical and practical implications

This study presents several theoretical and practical implications. From a theoretical perspective, this research advances the existing carbon emission reduction game model, which is based on a reward and punishment mechanism. It further incorporates a reputation mechanism and collusive behavior, thereby enriching evolutionary game theory and related models. Additionally, this model takes into account the collusive behavior of carbon verification agencies, thereby broadening the scope of stakeholder interactions in carbon emission reduction. This enhancement contributes to the theoretical framework of the study and has several practical implications. Firstly, it clarifies the relevant pathways for carbon emission management from a micro perspective, offering significant insights for the collaborative management of carbon emissions among the government, carbon verification agencies, and power enterprises. Secondly, the findings of this study provide valuable insights into the management of carbon verification agencies and the reduction of carbon emissions by enterprises. In the actual emission reduction process, it is essential to effectively utilize the government’s regulatory role, implement appropriate rewards and penalties for stakeholders involved in carbon emission reduction, and strengthen efforts against collusive behaviors. These measures are crucial for promoting fairness and justice in carbon emission reduction efforts.

### Limitations and future directions

This paper employs both theoretical and numerical analysis methods to investigate the behavior of carbon verification agencies and power enterprises in reducing low carbon emissions under government regulation, ultimately drawing several valuable conclusions. However, the paper does have some limitations. First, regarding data, due to constraints in data availability, this study relies on numerical simulations to validate the accuracy of theoretical derivations. While this serves as a validation method, integrating empirical research with numerical methods would strengthen the research conclusions. Second, concerning the research content, although this paper examines the low carbon emission reduction processes of carbon verification agencies and power enterprises, including aspects such as collusion, it is important to note that low carbon emission reduction is a complex behavior involving multiple stakeholders. Future research should also concentrate on critical behaviors, including the disclosure of information regarding low carbon emission reduction, the falsification of carbon data, and preferences for low carbon options. All of these factors are essential for effective reduction of carbon emissions.

### Conclusion

The authentic verification of carbon verification organizations significantly impacts China’s efforts to reduce carbon emissions. This paper examines the low-carbon dynamics between third-party carbon verification agencies and power enterprises under government regulation, considering factors such as government oversight, reputation mechanisms, costs, and collusive behavior. Utilizing evolutionary game theory and replicated dynamic equations, we construct a mathematical model to analyze the mechanisms influencing the carbon emission reduction strategies of carbon verification agencies and power enterprises across various scenarios. Our numerical simulation analysis reveals that the carbon emission reduction behaviors of these entities can be categorized into three distinct scenarios based on varying parameter conditions. Within a specific range, increasing government incentives for authentic verification by carbon verification agencies and for carbon emission reductions by power enterprises, as well as enhancing penalties for fraudulent verifications and failures to implement emission reductions, can yield positive outcomes. Furthermore, increasing the reputational losses associated with negative behaviors, reducing the collusive gains for both parties, and lowering the costs of low-carbon information disclosure and carbon emission reductions for power enterprises will encourage carbon verification organizations to adopt authentic supervision strategies and motivate power enterprises to pursue carbon emission reduction initiatives. This study aims to provide valuable references and insights to support the Chinese government’s efforts in promoting carbon emission reductions.

## Data Availability

If readers have a need for the research data in this paper, they can contact the corresponding author directly.
